# N-doped graphene foam obtained by microwave-assisted exfoliation of graphite

**DOI:** 10.1038/s41598-021-81769-5

**Published:** 2021-01-21

**Authors:** Malgorzata Skorupska, Anna Ilnicka, Jerzy P. Lukaszewicz

**Affiliations:** 1 Faculty of Chemistry, Nicolaus Copernicus University in Torun, Gagarina 7, 87-100 Torun, Poland; 2 Centre for Modern Interdisciplinary Technologies, Nicolaus Copernicus University in Torun, Wilenska 4, 87-100 Torun, Poland

**Keywords:** Materials chemistry, Surface chemistry

## Abstract

The synthesis of metal-free but electrochemically active electrode materials, which could be an important contributor to environmental protection, is the key motivation for this research approach. The progress of graphene material science in recent decades has contributed to the further development of nanotechnology and material engineering. Due to the unique properties of graphene materials, they have found many practical applications: among others, as catalysts in metal-air batteries, supercapacitors, or fuel cells. In order to create an economical and efficient material for energy production and storage applications, researchers focused on the introduction of additional heteroatoms to the graphene structure. As solutions for functionalizing pristine graphene structures are very difficult to implement, this article presents a facile method of preparing nitrogen-doped graphene foam in a microwave reactor. The influence of solvent type and microwave reactor holding time was investigated. To characterize the elemental content and structural properties of the obtained N-doped graphene materials, methods such as elemental analysis, high-resolution transmission electron microscopy, scanning electron microscopy, and Raman spectroscopy were used. Electrochemical activity in ORR of the obtained materials was tested using cyclic voltamperometry (CV) and linear sweep voltamperometry (LSV). The tests proved the materials’ high activity towards ORR, with the number of electrons reaching 3.46 for tested non-Pt materials, while the analogous value for the C-Pt (20 wt% loading) reference was 4.

## Introduction

Graphene possesses excellent mechanical and thermal properties^1–3^ and a high theoretical specific surface area (2630 m^2^ g^−1^)^4^. Therefore, over the past decades, graphene’s potential uses have been examined within many fields^5^: fields emission (FE) devices^6^, sensors and biosensors^7^, optoelectronic devices (transparent electrodes for solar cells), liquid–crystal displays (LCD)^8,9^, and electrode materials in batteries and supercapacitors^10,11^. Graphene can exist in the form of zero-dimensional (0D) dots (nanospheres^12^, hollow spheres^13^), one-dimensional (1D) fibers (ribbons)^14^, two-dimensional (2D) sheets^15^, and three-dimensional (3D) foams^16^. 3D graphene foam is a network of connected graphene sheets and, just as 2D graphene, can be widely used to improve the electrical, mechanical, or thermal properties of electrochemical devices. The size, quality, and type of graphene sheet synthesis determines the properties and potential application of the 3D materials^17^. The synthesis of a three-dimensional graphene structure is based on either the assembly of two-dimensional graphene flakes or on a direct synthesis. Many papers describe 3D structure synthesis based on chemical vapor deposition (CVD) of graphene on a nickel foam matrix^18–21^. A relatively new synthesis method is the 3D printing of graphene materials^22–24^. Well-known methods of synthesis produce three-dimensional graphene materials effectively, but they are too expensive and require specialized equipment. The assembly of graphene flakes^25,26^ by the hydrothermal method^27^ is also popular, although not without disadvantages; self-organization of graphene sheets is the most common problem that researchers want to eliminate^28^. The microwave method^29,30^ supports the synthesis of graphene, combining many advantages: it is fast, simple, does not require specialized equipment, and the starting material for foam production can be expanded graphite^31^ or sucrose^32^.

Graphene foams, due to their excellent porous structure, are a light material that combines the properties of two-dimensional graphene sheets and the three-dimensional system they form when joined together. The large pore volume and subsequent high specific surface area of the material, its thermal stability, high electronic conductivity, and high ion transfer rate all contribute to graphene foams’ functionality in energy storage devices^28,33,34^. Hence, to further improve their properties, increase electrode capacity, and power or energy density, many researchers try to dope graphene materials with metal compounds or metal oxides, or to create composites with other active materials^35–38^. These solutions involve increased production and disposal costs. The fact that these substances are not environmentally friendly leads researchers to try to introduce heteroatoms (mainly nitrogen) into the carbon structure using natural reagents^39,40^, thus increasing the range of potential applications of 3D graphene materials.

The synthesis of any metal-free but electrochemically active electrode material is an important contribution to protecting the environment and the key motivation for engaging this research approach. To this end, we present a fast and facile method of producing N-doped graphene foam using a microwave process. In the proposed process, expanded graphite was used as a starting material and green algae *Chlorella vulgaris* as an N-precursor. Different solvents (ethyl alcohol and dimethylformamide) were applied in the microwave reactor study. After the microwave process, the samples were carbonized. The study investigated the influence that the microwave (duration time) and carbonization processes had on graphene structure and nitrogen content.

## Results and discussion

### Material characterization

**Figure 1:**
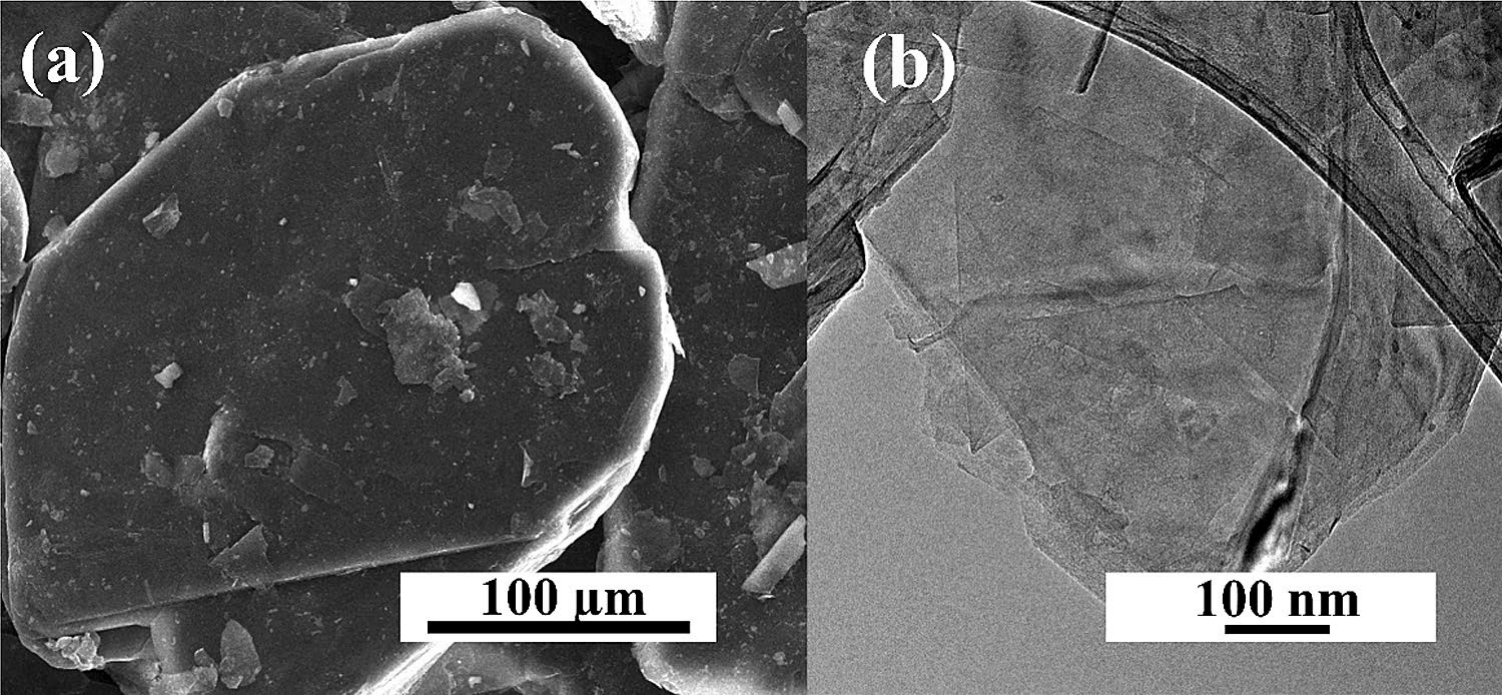
(**a**) SEM and (**b**) HRTEM images of expanded graphite.

**Figure 2:**
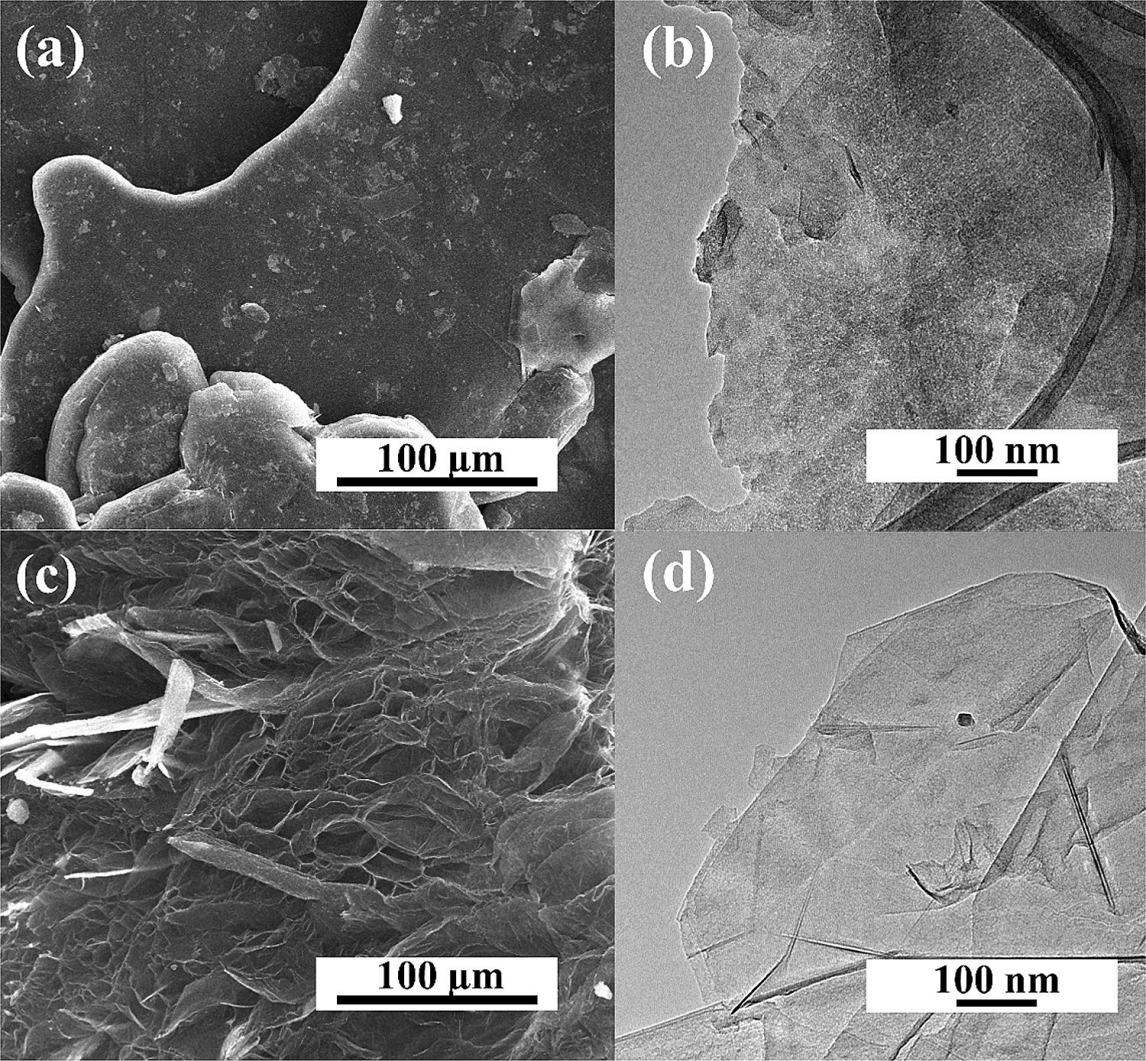
SEM and HRTEM images of graphene foams obtained without and with N-reagent: (**a**,**b**) 0A-10 and (**c**,**d**) 1A-10.

**Figure 3:**
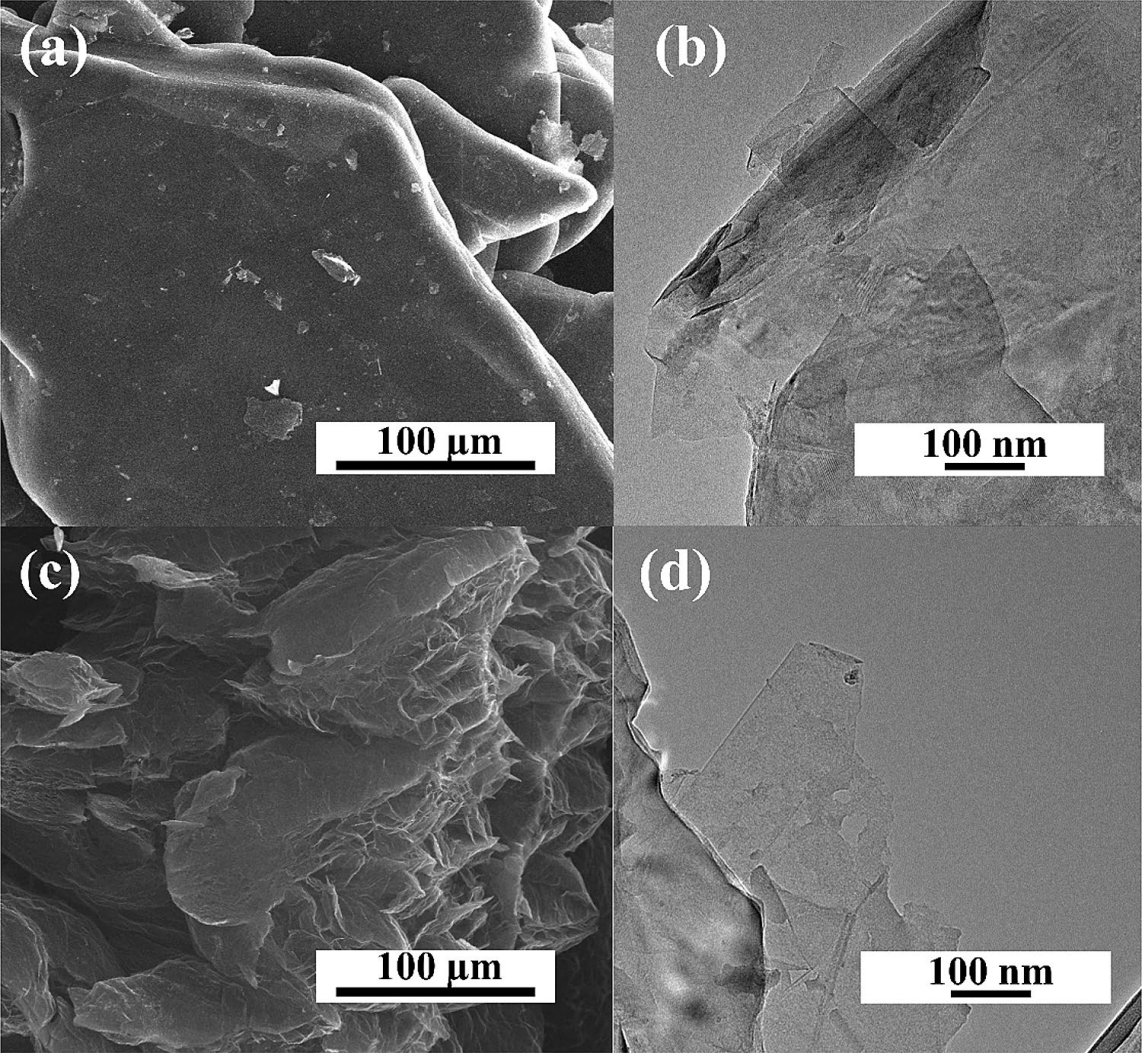
SEM, HRTEM images of graphene foams obtained without and with N-reagent: (**a**,**b**) 0B-10 and (**c**,**d**) 1B-10.

The proposed method to obtain N-doped graphene foam using a pressure microwave reactor was confirmed to be effective using instrumental methods. Figure 1 shows the commercial expanded graphite, the substrate for synthesis. The morphology of expanded graphite is flat and graphite sheets overlap in stacks. The scanning electron microscope images allow determining the impact of the microwave process and type of solvent on the structure of the obtained graphene materials. Figure 2 shows the structure of materials obtained in the EtOH solution for samples 0A-10 and 1A-10. For material obtained without the carbonization process, the structure is similar to pristine graphite with the layers not separated (Fig. 2a). The HRTEM images in Fig. 2b indicate that the material consisted of many graphene sheets forming thick stacked layers. Figure 2c,d show SEM and HRTEM images of graphene foams obtained with N-reagent. The carbonization process aided the exfoliation of graphene flakes. The SEM and HRTEM images for samples obtained in DMF are presented in Fig. 3a,b for the 0B-10 sample and Fig. 3c,d for the 1B-10 sample. The morphology of the materials obtained in DMF is very similar to that of materials obtained in EtOH, so further analysis was necessary. In all solvents, the material was exfoliated after carbonization to form graphene foam, creating very light and fluffy structures. The high temperature caused the creation of spaces and a system of very thin graphene walls which incorporated nitrogen atoms. The HRTEM images for all solvents indicate that in both cases very thin parallel layers can be observed. Disordered morphology and clearly overlapping layers are typical for graphene-based materials.

A basic premise of this method of enriching the final material with nitrogen is the use of green algae. After carbonization, it can join graphene flakes (single layer graphene, few layer graphene and multilayer graphene type) into a durable, porous 3D structure resembling a foam or sponge. Aside from providing amorphous carbon matter adhering graphene flakes, green algae is a source of nitrogen. It was selected due to its useful features, such as ease of mixing with graphene flakes in EtOH or DMF and thermal degradation to a non-porous nitrogen-rich matrix. A low carbon matrix yield from the starting green algae mass makes it possible to avoid soaking the graphene flakes and retains the graphene flakes’ surface accessibility to chemical reagents, e.g., electrolytes. The green algae-originated carbon phase is assumed to be present in the form N-rich bridges between graphene flakes which enable electron transfer throughout the whole mass of material. The effective exfoliation of materials obtained with a holding time of 10 min in the reactor led to the investigation of the effect that extending holding time had on the structure and elemental composition of graphene foams. The influence on the nitrogen content was examined in two different electrolytes, while the carbonization process was used to change the structure of graphene by creating exfoliated graphene foam with embedded nitrogen functional groups. The elemental analysis was used to determine the composition of the N-doped graphene foams. The influence of the elemental composition for materials in series 0A/B-T and 1A/B-T obtained in different solvents is shown in Table 1. The results for samples obtained in these two series indicate that solvent and holding time in the microwave reactor influence the elemental content of the obtained materials. A high carbon content in the range from 76.06 to 95.52 wt% was observed for all samples. The mass content of the samples obtained was typical for graphene-based materials. The nitrogen content in samples prepared without N-reagent was significantly lower than that for samples with *Chlorella vulgaris*. Heteroatoms were successfully introduced into the graphene structure using the natural reagent. For samples synthesized in different solvents with N-reagent, the percentage by mass of nitrogen was high, at 2.44 wt% for the 1A-10 sample. Along with extending the reaction time for the 1A-T series produced in the ethyl alcohol solvent, a significant decrease of the nitrogen content, down to 0.65 wt%, was noted. In the case of the 1B-T series, where DMF was the solvent, an increase in the samples’ nitrogen content was observed when holding time was increased. The highest nitrogen content for the 1B-T series was recorded for a sample maintained for 90 min, at 0.89 wt%.

**Table 1:** Elemental composition of EG and graphene foams obtained with different solvents and holding times in the microwave reactor.

Sample	Content (wt%)		
	C	H	N
EG	73.54	1.50	0.08
0A-10	76.06	1.56	0.14
0B-10	80.70	1.37	0.58
1A-10	88.83	0.58	2.44
1A-30	90.40	0.61	0.97
1A-60	91.09	0.90	0.65
1A-90	91.50	0.83	0.73
1B-10	95.48	0.34	0.10
1B-30	91.27	0.72	0.76
1B-60	89.21	0.92	0.84
1B-90	89.78	0.83	0.89

**Figure 4:**
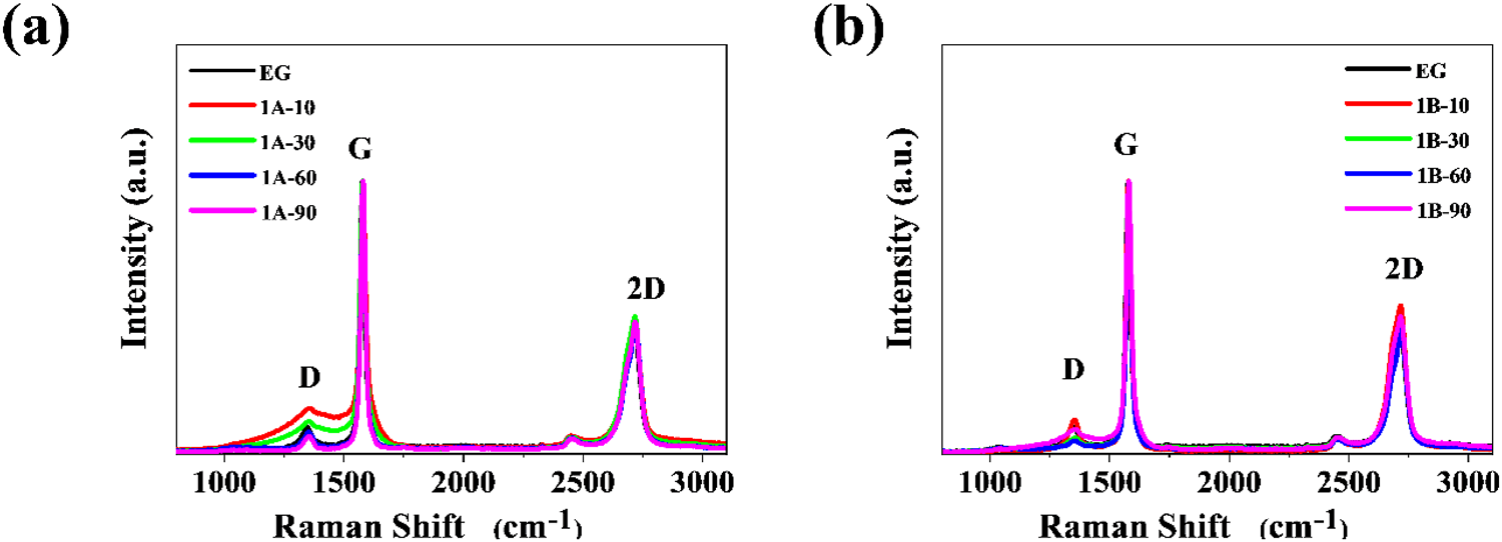
Raman spectra of (**a**) 1A-T and (**b**) 1B-T series compared to pristine EG.

Raman spectroscopy is the method most commonly used to characterize graphene materials^41^. On the Raman spectra for graphene, three characteristic bands can be observed, D, G, and 2D, for which the shift at the 532 nm laser line is 1347 cm^−1^, 1577 cm^−1^, and 2698 cm^−1^^42^ respectively. Using Raman spectroscopy analysis, it is possible to estimate the number of graphene sheet layers that have been laid, as well as determine defects in the material obtained. Raman spectra were compiled for the N-doped graphene foams carried out in two selected solvents, EtOH (Fig. 4a) and DMF (Fig. 4b). The holding time in the reactor was 10, 30, 60, or 90 min. Table 2 shows the ratio of intensities of the materials obtained depending on their different reaction times and solvents. The ratio of D and G band intensities indicates the degree of graphitization defect, while I_2D_/I_G_ indicates the number of superimposed graphene layers. Comparing the spectra with each other, there is no significant difference in the intensity ratio of 2D to G bands. The ratio is close to 0.5, which indicates that the obtained material possesses several layers. The change in D-band is clearly visible, which may mark the quality of the graphene structure. In the 1A-T series, the I_D_/I_G_ band ratio increases along with the reaction time, indicating quality. The low ratio of D to G-band intensities suggests that graphene material was effectively obtained. These can potentially be used in electrochemical applications as electrodes, as sensors, or biosensors.

**Table 2:** Raman band positions and intensities, as well as the intensity ratio of EG and graphene obtained in 1A-T and 1B-T series.

Sample	D-band		G-band		2D-band		I_D_/I_G_	I_2D_/I_G_
	cm^−1^	I	cm^−1^	I	cm^−1^	I		
EG	1348.0	0.10	1575.5	1	2713.5	0.48	0.10	0.48
1A-10	1353.0	0.17	1580.0	1	2716.0	0.47	0.17	0.47
1A-30	1353.0	0.12	1578.5	1	2716.0	0.50	0.12	0.50
1A-60	1352.5	0.08	1580.0	1	2718.5	0.48	0.08	0.48
1A-90	1353.0	0.07	1580.0	1	2717.5	0.48	0.07	0.48
1B-10	1353.0	0.12	1578.5	1	2716.0	0.55	0.12	0.55
1B-30	1355.5	0.06	1580.0	1	2717.5	0.49	0.06	0.49
1B-60	1353.0	0.05	1580.0	1	2719.0	0.47	0.05	0.47
1B-90	1356.5	0.09	1580.0	1	2715.5	0.50	0.09	0.50

**Figure 5:**
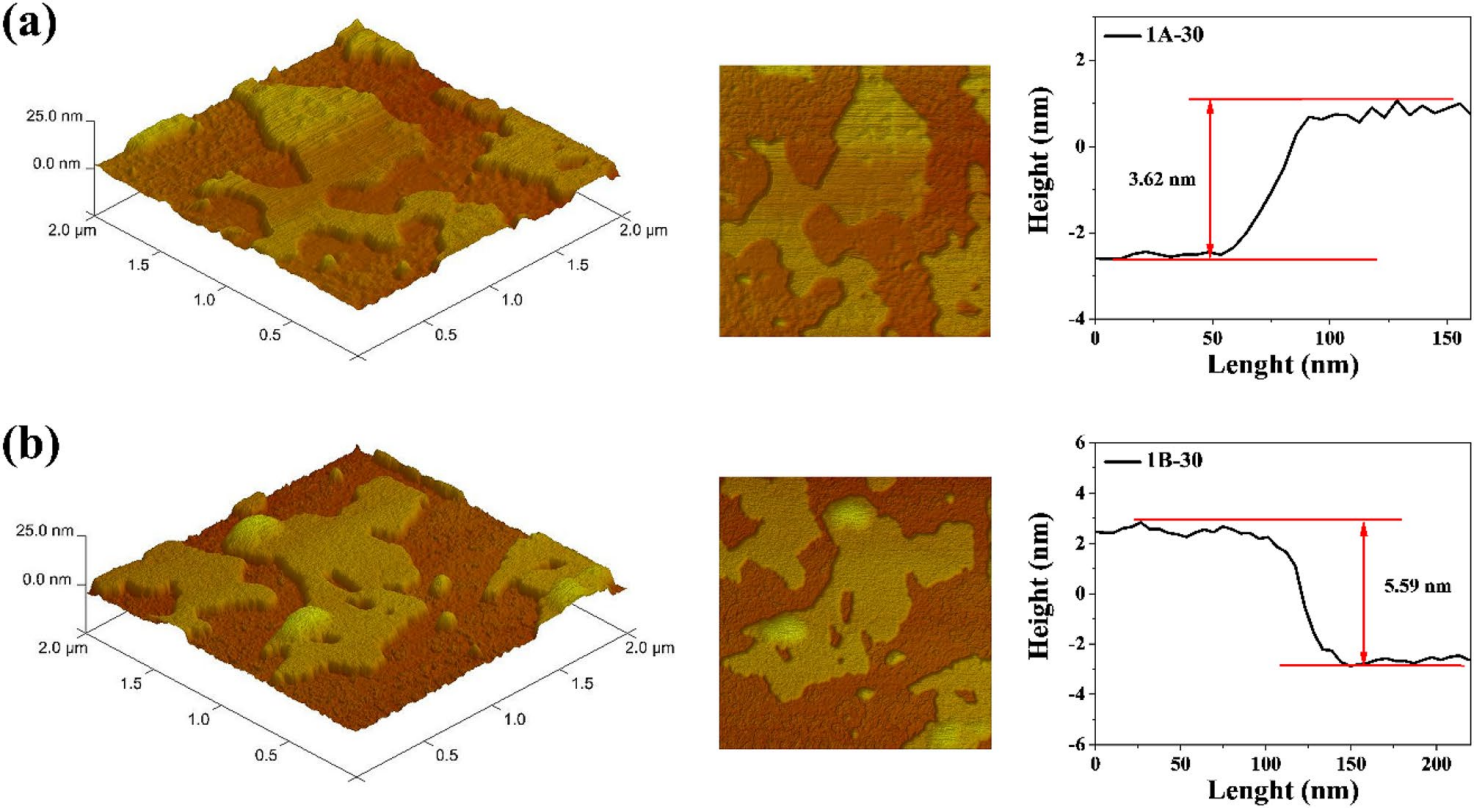
AFM images of (**a**) 1A-30, (**b**) 1B-30 with height profiles of graphene flakes.

In order to determine the thickness and lateral size of graphene sheets which were treated in the microwave reactor, AFM analysis was performed using the 1A-T and 1B-T series of samples, synthesized in two solvents, EtOH (Fig. 5a) and DMF (Fig. 5b). Knowing the thickness of single-layered graphene and the interlayer distance, the measured value corresponds to few graphene layers. The thickness of graphene sheets for series 1A-T and 1B-T is in the range of 3.62–6.78 nm and 5.59–16.45 nm, respectively. This result is consistent with Raman spectroscopy examinations, which also suggests that employing the microwave reactor produces few-layered graphene, regardless of the type of solvent.

**Figure 6:**
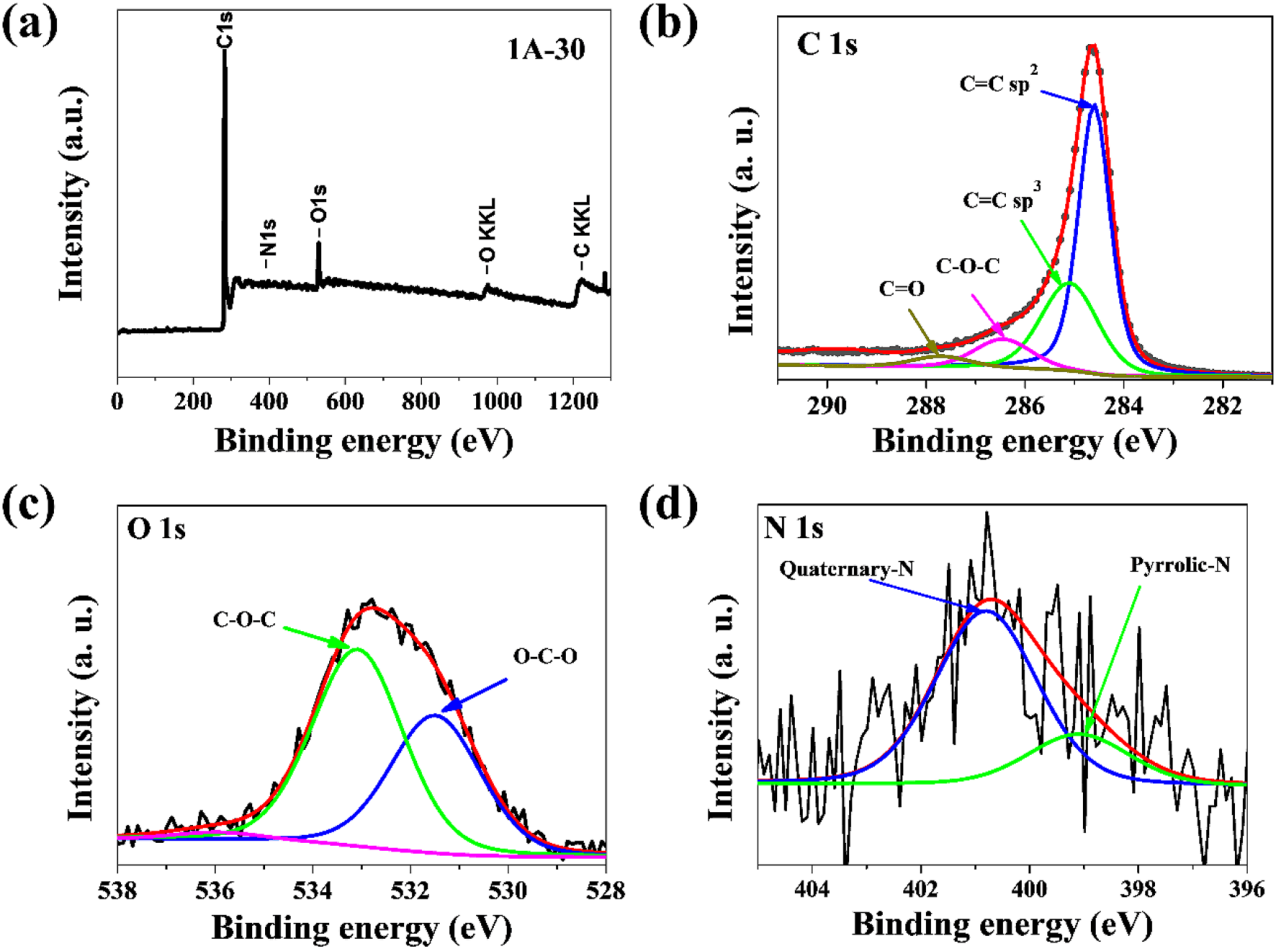
(**a**) XPS survey spectrum and high-resolution XPS spectra of (**b**) C1s, (**c**) O1s, (**d**) N1s for 1A-30 sample.

The surface elemental composition of materials from the 1A-T series was analyzed using X-ray photoelectron spectroscopy. Figure 6a shows a wide survey scan for sample 1A-30 collected from 0 to 1300 eV. XPS spectra of sample 1A-30 for C1s, N1s, and O1s are presented in Fig. 6b–d. The high-resolution spectrum of C1s (Fig. 6b) can be deconvoluted into five peaks centered at 284.6 eV, 285.0 eV, 286.4 eV, 287.7 eV, and 288.6 eV, which can be attributed to bonds of C=C (sp^2^), C–C (sp^3^), C–O–C, C=O, and O–C=O, respectively. The total content of carbon for all samples, determined by XPS measurement, was in the range from 90.2 to 93.2 at.% (Table 3) and was at the same level as carbon content determined by elemental combustion measurement.

**Table 3:** Chemical composition analyzed using high-resolution XPS for series 1A-T.

Sample	Binding energy (eV)									
	284.6	285.0	286.4	287.7	288.6	531.6	533.2	536.0	399.1	400.8
	Elemental content (at.%)									
	C					O			N	
1A-10	35.7	41.3	9.3	3.0	0.9	2.7	4.5	0.2	0.9	0.8
1A-30	57.6	24.0	7.9	2.9	0.0	2.5	3.5	0.1	0.7	0.2
1A-60	66.5	16.0	7.9	2.6	0.0	2.4	3.2	0.1	0.5	0.3
1A-90	59.9	23.5	7.1	2.7	0.0	2.4	2.9	0.3	0.4	0.2

After deconvolution of the high-resolution spectrum of N1s, two characteristic types of bonds were determined for nitrogen in the form of pyrrolic-N and quaternary-N in binding energies of 399.1 eV and 400.8 eV, respectively^43^. Modified graphene structures subjected to microwave heating facilitated nitrogen substitution in the graphite plane. The effect of high carbonization temperatures on doped structures was the formation of mainly quaternary-N functional groups, responsible for the materials’ effective catalytic activity in the oxygen reduction reaction^44^. Due to access to an electron pair in the graphene structure, the pyrolytic N-group is active in response to the oxygen reduction reaction, which leads to an increase in the catalytic activity of N-doped materials^45^. The overall content of nitrogen atoms for all samples in the 1A-T series was between 0.6 and 2.7 at.%. The XPS results show that nitrogen doping in graphene is successful; there were two characteristic types of nitrogen functional groups that improved catalytic properties in response to ORR results, the highest content belonging to the pyrrolic-N groups. Deconvolution of the high-resolution O1s spectrum fit into three bands at energy values of 531.6 eV, 533.2 eV, and 536.0 eV which correspond to C–O–C, O–C–O, and absorbed H_2_O bonds, respectively^46^.

### Electrochemical performance

**Figure 7:**
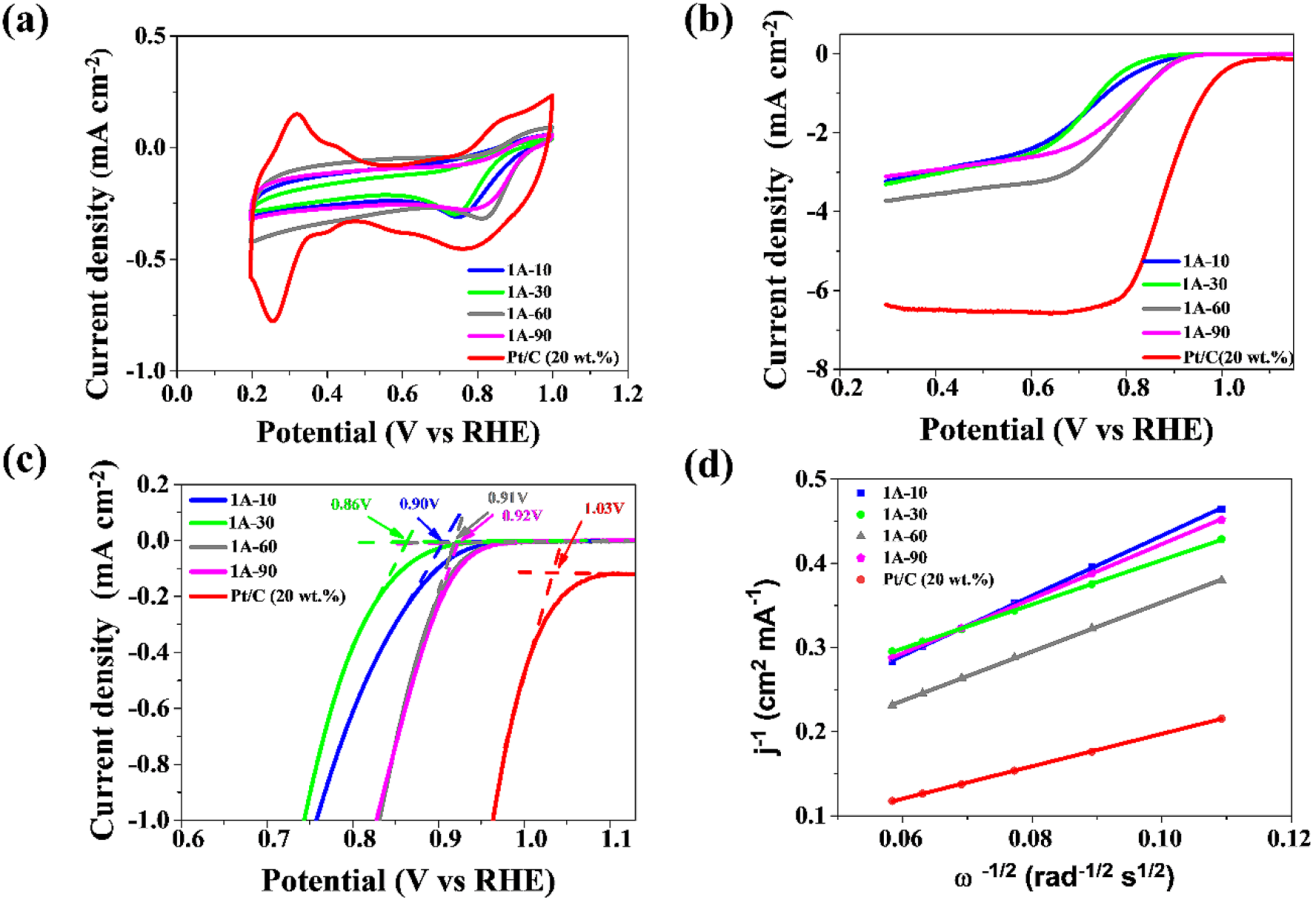
(**a**) CV curves with a scan speed of 100 mV s^−1^ in O_2_-saturated electrolyte for the 1A-T series and the Pt/C catalyst; (**b**) LSV curve of the obtained samples and Pt/C catalysts (5 V s^−1^, 1600 rpm); (**c**) Onset potential of 1A-T and the commercial catalyst; (**d**) Koutecky–Levich plot for the samples at 0.45 V.

In order to check the catalytic activity of the obtained materials in the oxygen reduction reaction, cyclic voltammetry and linear voltammetry measurements were taken. The ORR reaction is distinctive in metal-air batteries and fuel cells, therefore examining the catalytic activity of the produced nitrogen-doped materials was an important aspect of the work. In recent years, heteroatom-doped graphene has become an alternative to platinum-based materials owing to the heteroatoms’ improvement of electrochemical properties^47,48^. In this case, graphene structures served as a matrix for the introduction of nitrogen, using the natural substrate *Chlorella vurgalis* as the source. As the literature reports, N-precursor is transformed into conductive carbon forms under the influence of high carbonization temperatures, 800–900 °C, which increase electrocatalytic properties^49,50^. The reference material in electrochemical tests was a carbon material based on platinum (20 wt%). All measurements were made in an oxygen-saturated 0.1 M KOH electrolyte for the materials from the 1A-T and 1B-T series, however the plots are presented for the 1A-T series only (Fig. 7). Figure 7a illustrates the CV curves for the samples obtained in the 1A-T series, showing the cathode peaks for samples 1A-10, 1A-30, 1A-60, and 1A-90 at a potential of 0.75 V, 0.74 V, 0.81 V, and 0.76 V relative to the RHE electrode, respectively. This is one proof that the presented method produces materials with good electrocatalytic properties. When examining the catalytic activity in the oxygen reduction reaction, one should keep in mind the measurements of linear voltammetry, which provide the most information regarding the catalytic properties of the obtained materials. Figure 7b shows LSV measurements. The curves were retrieved with oxygen flow at a scanning speed of 5 mV s^−1^, and rotation speed of 800–2800 rpm. A curve was selected for all samples in the 1A-T series and for the Pt/C reference catalyst (20 wt%) at one rotation speed, 1600 rpm. Sample 1A_60 exhibits the highest current density among the samples obtained, but it is still lower than a commercial material based on platinum. This proves that, despite sample 1A-60 having the lowest nitrogen content at 0.65 wt%, the material shows good catalytic properties in ORR. The value of the onset potential for the 1A-60 sample, shown in Fig. 7c, was 0.91 V, while for the commercial material this value was about 1.03 V. For the other samples, 1A-10, 1A-30, and 1A-90, the value of the onset potential was 0.90, 0.86, and 0.92 V vs RHE, respectively. LSV results are typical for non-metallic graphene-based materials. They contributed to the estimation of the electron transfer number in the oxygen reduction reaction, which primarily describes the path of the ORR reaction. By analyzing the Koutecky–Levich plot (Fig. 7d), it is possible to determine the number of electron transfers. Data gathered from the LSV plot at a value of 0.45 V were used to calculate the number of electrons participating in the reaction. For the commercial material based on platinum, a four-electron reaction was a distinguishing characteristic, while graphene is characterized by a two-electron oxygen reduction reaction^51^. ORR performance parameters for the 1A-T series and commercial platinum-based carbon are presented in Table 4. The values closest to those for the commercial catalyst were those of samples 1A-30 and 1A-60, of 3.46 and 3.10, respectively. A high value of n means that the electron transfer in the oxygen reduction reaction is close to the 4 electron ORR mechanism. The number of transferred electrons for the series of samples obtained with DMF as solvent equals 2.55, 2.42, 2.49, and 2.57 for 1B-10, 1B-30, 1B-60, and 1B-90, respectively. The produced materials show improved properties over pristine graphene, whose typical number of electron transfers in ORR is about 2 electrons^48,52^. The LSV results show that the resulting materials have good catalytic properties. This is proof that the electrocatalysts doped with heteroatomic nitrogen, and mainly nitrogen functional groups in the graphene structure, are responsible for catalyzing the ORR reaction. The products are completely free of any metals and can potentially achieve wide application in the field of electrochemistry. Remarkably, the 1A-60 and 1A-90 exhibited prominent ORR activity with the most positive onset potential (E_onset_) at 0.91 V and 0.92 V. The half-way potential (E_1/2_**)** was also highest for 1A-60 and 1A-90 compared to other samples. The prominent ORR activities of 1A-30 and 1A-60 in an alkaline medium may be benefitting from the synergistic effect of the structure and elemental composition. First, the unique few-layer architecture of graphene is conducive to exposing more of the active sites and facilitates the accessibility of oxygen. Second, the nitrogen heteroatoms can induce charge redistribution and facilitate the adsorption of oxygen and the consequent reduction reaction on carbon. The proposed materials’ composition is their advantage, because generally structures described for ORR application tend to be decorated with expensive, precious, or environmentally-unfriendly metals or metal oxides, e.g., Pd, Ru^53^, perovskie oxides^54^, Fe(II)^55^, Fe_7_C_3_^56^_,_ or Au^57^.

**Table 4:** ORR performance parameters of series 1A-T and commercial Pt/C catalyst tested in alkaline media.

Sample	E _onset_ (V vs RHE)	E_1/2_ (V vs RHE)	Diffusion limiting current density (mA cm^−2^)	n (0.45 V)
1A-10	0.90	0.73	2.56	2.55
1A-30	0.86	0.72	2.44	3.46
1A-60	0.91	0.79	3.55	3.10
1A-90	0.92	0.80	3.12	2.80
Pt/C (20 wt%)	1.03	0.88	6.37	4.00

## Conclusion

In summary, an effective method of obtaining N-doped graphene in a microwave reactor was developed, one which uses expanded graphite and *Chlorella vurgalis*. Research indicates that the use of a natural N-reagent during synthesis in a microwave reactor in various solvents can contribute to the incorporation of nitrogen atoms into the graphene structure at a level of several percent. It can be concluded that ethyl alcohol and dimethylformamide are good solvents which contribute to the exfoliation of graphene layers and the production of pure graphene material. The high quality of the obtained material, without defects, shows that the method is a promising way of producing heteroatom-doped graphene foams. The optimal time for synthesis in the microwave reactor was 30 min and introduced mainly pyrrolic-N groups descend from N-precursor *Chlorella vurgalis*. The best electrochemical results were obtained for materials using ethanol as a reaction medium. The solvent also influenced the introduction of nitrogen functional groups into the graphene structure. The products showed good catalytic activity owing to the nitrogen functional groups introduced into the graphene structure, which are responsible for the catalytic properties in the oxygen reduction reaction. The tests proved their high activity towards ORR, i.e., the number of electrons n reached 3.46 for tested materials (no Pt added), while the analogous value for the C-Pt (20 wt% loading) reference was only slightly higher and equal 4. For pristine, i.e., non-N-doped graphene, the n value was only 2. It has to be underlined that the progress in electrochemical performance was achieved in an environment-friendly way, not only without the use of noble metals, but also with a reduction in energy consumption. Heteroatom doping of graphene materials is an alternative to more expensive materials based on metals, an alternative that can have promising properties for electrochemical applications and a huge impact on the potential use the composite materials in supercapacitors, metal-air batteries, or fuel cells.

## Materials and methods

### Preparation of N-doped graphene foam samples

The production of N-doped graphene foams was based on a one-step process using microwave radiation in two dispersed solvents: ethyl alcohol (EtOH), and dimethylformamide (DMF). The starting materials used were expanded commercial graphite (EG, Sinograf, Torun, Poland) and the natural green algae N-precursor, *Chlorella vulgaris*. The compounds were mixed in a solvent with a 1:4 mass ratio of graphene to *Chlorella vulgaris*, then sonicated to obtain a better dispersion. The samples were loaded into a microwave reactor (Microwave 400) and heated under pressure to 180 °C, with the power of about 300–600 W. The microwaving duration was either 10, 30, 60, or 90 min; after this time, the samples were stirred. The obtained mass was then washed with distilled water to remove the solvent and dried at 120 °C overnight. Finally, samples were carbonized at 900 °C with a heating rate of 3 °C min^−1^ and then heated for 4 h under the flow of nitrogen (to provide an inert environment during carbonization). Reference samples were made using the same procedure, but without the addition of an N-precursor nor the carbonization process. For all samples without the addition of *Chlorella vulgaris*, the process in the reactor was maintained for 10 min. The reference samples obtained without carbonization, only the microwave process, are designated as 0A/B-T, where A and B describe the solvent and denote EtOH or DMF, respectively, while T is the time in minutes that the sample was held in the microwave reactor. Samples obtained with the addition of the N-precursor and using carbonization are designated as 1A/B-T, where A and B describe the solvent and mark EtOH or DMF, respectively, and T is the duration of time spent in the microwave.

### Characterization methods

The atomic structure of the obtained materials was analyzed using a high-resolution transmission electron microscope (HRTEM FEI Tecnai F20 X-Twin, Brno, Czech Republic) at an accelerating voltage of 200 kV. A scanning electron microscope operating at 30 kV (SEM, 1430 VP, LEO Electron Microscopy Ltd., Oberkochen, Germany) was used to determine the structure of the samples. The elemental composition of the materials was analyzed by means of a combustion elemental analyzer (Vario MACRO CHN, Elementar Analysensysteme GmbH, Langenselbold, Germany). Raman-microscopic spectroscopy analysis was performed using Renishaw InVia (Renishaw Company, Gloucestershire, the United Kingdom), excitation wavelength 532 nm at an ambient temperature. Nitrogen sorption isotherms were determined through nitrogen physisorption at 77 K in a volumetric apparatus ASAP 2010 (Micromeritics, Norcross, GA, USA). The attachment, bonding configuration, and compositional analysis of the material was carried out by X-ray photoelectron spectroscopy measurements (XPS, PHI5000 VersaProbe II Scanning XPS Microprobe, Chigasaki, Japan). A monochromatic Al-Kα X-ray (1486.6 eV) was used as the operating excitation source. Atomic Force Microscopy (AFM) investigations were conducted using a Scanning Probe Microscope (SPM) apparatus produced by Veeco (Digital Instrument, USA), working under ambient conditions.

### Electrochemical measurements

Tests of the obtained materials’ electrochemical properties were carried out using the Autolab potentiostat (PGSTAT128N, The Netherlands). The standard measuring setup was made up of three electrodes—an Ag/AgCl electrode in 3 M KCl was the reference, a Pt electrode the counter, while the working electrode was glassy carbon (GC rotating disk electrode with a 5.0 mm diameter) onto which ink, with the catalyst, was applied. The catalyst ink was prepared basing on the dispersion of 2.5 mg of the test sample in a mixture of distilled water and ethanol (ratio 1:4) and Nafion (0.5 wt% Nafion). As a point of comparison, electrochemical studies were also performed on a commercial catalyst from Sigma Aldrich, platinum on graphitized Pt/C carbon (20 wt% Platinum). The prepared ink was pipetted onto glassy carbon in an amount of 15.63 µl and the electrode was dried afterwards. Catalyst content was about 0.4 mg cm^−2^. Before electrochemical testing, the alkaline solution was saturated with O_2_/N_2_ for 20 min. The activity of the samples in an oxygen reduction reaction was evaluated by measuring cyclic voltammetry (CV), carried out at a scanning speed of 10 mV s^−1^, and linear voltammetry (LSV), at a scanning speed of 5 mV s^−1^ and rotation speed of 800–2800 rpm, in a solution of 0.1 M KOH at room temperature. The number of electrons (n) directly participating in ORR was calculated using the Koutecky–Levich equation (K–L):

1 $${\text{J}}^{{ - {1}}} = {\text{ J}}_{{\text{L}}}^{{ - {1}}} + {\text{ J}}_{{\text{K}}}^{{ - {1}}} = \, \left( {{\text{B}}\omega^{{{1}/{2}}} } \right)^{{ - {1}}} + {\text{ J}}_{{\text{K}}}^{{ - {1}}}$$

2 $${\text{B }} = \, 0.{\text{62nFC}}_{0} \left( {{\text{D}}_{0} } \right)^{{{2}/{3}}} \nu^{{ - {1}/{6}}}$$

where J is defined as the measured density, J_L_ is the current density limiting diffusion, and J_K_ is the kinetic current density; ω is the angular velocity of the electrode; n is the number of electron transfers in the reaction; F is the Faraday constant (96,485 C mol^−1^); C_0_ is the concentration of dissolved oxygen (1.2 × 10^–6^ mol L^−1^ in 0.1 M KOH); D_0_ is the diffusion coefficient of dissolved oxygen (1.9 × 10^–5^ cm^2^ s^−1^ in 0.1 M KOH); ν is the kinetic viscosity of the electrolyte for 0.1 M KOH, 0.01 cm^2^ s^−1^. The slope of the K-L graph can be obtained using Koutecky–Levich Eqs. (1) and (2), thanks to which it is possible to determine the number of electron transfers in ORR.
